# Assessing neonatal nurses: transitioning preterm infants to oral feeding - a multicenter cross-sectional study

**DOI:** 10.1186/s12912-024-02647-9

**Published:** 2025-01-09

**Authors:** Abdelaziz Hendy, Yasmine M. Osman, Hanan F. Alharbi, Maha Suwailem Shuaib Alshammari, Mohammed Musaed Ahmed Al-Jabri, Naif S. Alzahrani, Ahmed Hendy, Abdulaziz Mofdy Almarwani

**Affiliations:** 1 Department of Pediatric Nursing, Faculty Nursing, Ain Shams University, Cairo, Egypt; 2 Department of Obstetrics and Gynecology Nursing, Faculty of Nursing, Zagazig University, Zagazig, Egypt; 3 Maternity and Pediatric Nursing Department, College of Nursing, Princess Nourah bint Abdulrahman University, Riyadh, Saudi Arabia; 4 Department of Medical Surgical Nursing, College of Nursing, Jouf University, Sakākā, Saudi Arabia; 5 Nursing Department, Critical Care Nursing, College of Applied Medical Sciences, Prince Sattam Bin Abdulaziz University, Wadi Aldawaser, Saudi Arabia; 6 Department of Medical-Surgical Nursing, College of Nursing, Taibah University, Medina, Saudi Arabia; 7 Department of Computational Mathematics and Computer Science, Institute of Natural Sciences and Mathematics, Ural Federal University, Yekaterinburg, 620002 Russian Federation; 8 Department of Mechanics and Mathematics, Western Caspian University, Baku, 1001 Azerbaijan; 9 Department of Nursing Administration and Education, College of Nursing, Taibah University, Medina, Saudi Arabia

**Keywords:** Neonatal nursing, Premature, Oral feeding, Feeding methods, Health knowledge, Attitude, Practice

## Abstract

**Background:**

In Egypt, approximately 10% of preterm deliveries occur between 32 and fewer than 37 weeks, leading to high neonatal intensive care unit (NICU) admissions. Preterm infants often face oral feeding difficulties due to immature development, which can lead to extended hospital stays and increased health risks.

**Aim:**

To assess neonatal nurses’ performance in terms of the transition to oral feeding in preterm infants, focusing on knowledge, practices, and attitudes.

**Methods:**

A descriptive, quantitative, multicenter, cross-sectional study was conducted across 16 hospitals in five governorates in Egypt from November 2023 to March 2024 involving 553 neonatal nurses. The data were collected through a self-administered questionnaire assessing knowledge and attitudes and through an observed checklist for nurses’ practices. The study used statistical methods, including binary logistic regression, to analyze the data.

**Results:**

The findings revealed significant knowledge gaps among nurses, particularly in terms of oro-motor function, suck-swallow-breathe patterns, and nonnutritive sucking. A total of 64.6% of the nurses had unsatisfactory knowledge, 58.6% had unsatisfactory practices, and 45% had a negative attitude toward the oral feeding transition. Key predictors of satisfactory practices included higher education levels, full-time employment, and positive attitudes.

**Conclusion:**

This study highlights critical gaps in neonatal nurses’ knowledge and practices regarding the transition to oral feeding in preterm infants. Addressing these gaps through targeted educational interventions and ongoing support is essential for improving care quality and outcomes for infants. The findings revealed that a substantial proportion of nurses lacked adequate knowledge of critical areas, such as oro-motor function, the suck-swallow-breathe pattern, and nonnutritive sucking. These knowledge deficits could hinder the ability of nurses to provide optimal care during this crucial transition.

**Supplementary Information:**

The online version contains supplementary material available at 10.1186/s12912-024-02647-9.

## Introduction

Prematurity, as defined by the World Health Organization (WHO), occurs when infants are born before completing 37 weeks of gestation or within 259 days from the start of the mother’s last menstrual period [1–3]. There are an estimated 13.4 million babies worldwide who are born prematurely each year [4, 5]. Approximately 10% of preterm deliveries occurring between 32 and fewer than 37 weeks of gestation are documented in Egypt. This significant difference may lead to a greater number of admissions to the neonatal intensive care unit (NICU) annually [6]. Prematurity is the leading cause of mortality and morbidity among children under the age of 5 globally. It is linked to numerous negative health outcomes that affect survival and quality of life later in life [7–9]. Preterm infants often experience significant oral feeding difficulties, primarily because of the underdevelopment of their central nervous system, cardiovascular and respiratory systems, and musculature of the mouth [10]; approximately 40–70% of preterm infants experience feeding difficulties. These challenges often result in prolonged hospital stays, as these infants require extended care to support their growth and development until they are stable enough for discharge [11–13].

Oral feeding is a complex and dynamic process vital for preterm infant growth and development during their stay in the NICU. This critical milestone requires the coordination of various systems, including the oral-motor, neurological, cardiorespiratory, and gastrointestinal systems. Efficient and safe oral feeding depends on the proper functioning and integration of these systems to ensure that the infant can feed effectively [14]. Preterm infants with immature oral feeding abilities can adversely affect their development and increase the risk for aspiration, hypoxemia, bradycardia, pneumonia, and even mortality [15]. Currently, the most significant issue concerning premature infants is the transition to oral feeding. Independent oral feeding is a critical milestone that must be achieved before hospital discharge [16]. Furthermore, achieving an effective transition to oral feeding could decrease the length of hospitalization, alleviate the family’s financial burdens and foster emotional bonding between parents [17].

The transition from tube feeding to full oral feeding in preterm infants is influenced by several factors, including the infant’s physiological stability and overall neurodevelopmental maturity. This maturity encompasses behavioral organization and the coordination of rhythmic sucking, swallowing, and breathing. Effective cardiorespiratory regulation is also critical. Additionally, specific infant characteristics, such as low birth weight, gestational age at birth, and overall neonatal condition, are crucial. These factors can guide healthcare professionals in determining the appropriate timing and method for introducing oral feeding to preterm infants [18].

As the primary caregivers in the NICU, neonatal nurses play an essential role in evaluating preterm infants’ readiness cues for beginning oral feeding [19]. To effectually support preterm infants’ transition to oral feeding, neonatal nurses need comprehensive knowledge about various areas, such as appropriate timing and feeding position, nonnutritive sucking, readiness for feeding, oral stimulation exercises and cue-based feeding [16]. Nurses training about evidence-based nursing interventions that include oral motor stimulation and nonnutritive factors helps preterm infants develop feeding skills and early transition to oral feeding [20]. Therefore, assessing nurses’ performance regarding the transition to oral feeding for preterm infants is the first step toward improving feeding difficulties for preterm infants.

Although several studies have evaluated the process of transition from tube to oral feeding in preterm infants and the implementation of evidence-based intervention protocols [21–24], there is a noticeable scarcity of research assessing the performance of neonatal nurses in relation to oral feeding for preterm infants. Thus, this study aimed to assess neonatal nurses’ performance in terms of the transition to oral feeding in preterm infants working in the neonatal intensive care unit.

Prematurity is a major health problem because it is a leading cause of infant morbidity and mortality in developing countries like Egypt [9]. Accurate data on the prevalence of prematurity is lacking due to Egypt’s poor recording system. However, in spite of existing guidelines and the implementations of evidence-based protocols regarding evaluating transitioning to the oral feeding process among premature neonates, it remains an ongoing challenge as the number of studies evaluating the knowledge of neonatal nurses in Egypt about the transition to oral feeding of preterm infants is limited. In addition, overzealous feeding practice and negative oral simulation may result in disorganization of the oro-motor function, which leads to feeding problems [21–24]. This is the first multi-central study conducted in Egypt to assess the knowledge and performance of the nurses regarding the transition to oral feeding in preterm infants working in the neonatal intensive care unit.

## Aim of the study

The performance of neonatal nurses regarding the transition to oral feeding among preterm infants was assessed in terms of knowledge, practices, and attitudes.

**Specific objectives**.

- To assess the knowledge of neonatal nurses regarding the transition to oral feeding in preterm infants.
- To examine the practices of neonatal nurses regarding the transition to oral feeding in preterm infants.
- To explore the attitudes of neonatal nurses regarding the transition to oral feeding in preterm infants.
- To evaluate the relationship between neonatal nurses’ performance in the transition to oral feeding in preterm infants and their sociodemographic characteristics.

### Research questions

1. What is the current level of neonatal nurses’ knowledge regarding the transition to oral feeding in preterm infants?
2. What is the level of neonatal nurses’ reported practice regarding the transition to oral feeding in preterm infants?
3. What is the level of neonatal nurses’ knowledge regarding the transition to oral feeding in preterm infants?
4. Is there a relationship between neonatal nurses’ performance in the transition to oral feeding in preterm infants and their sociodemographic characteristics?

## Methods

### Study Design

The descriptive, quantitative, multicenter cross-sectional design involves the systematic collection, analysis, and interpretation of data to provide a clear picture of a particular situation; this process was carried out in 16 hospitals to assess neonatal intensive care unit nurses’ performance in terms of the transition to oral feeding in preterm infants in terms of knowledge, practice, and attitude.

### Sampling and recruitment

A convenience sample of all the available neonatal nurses working in the NICU was selected. The inclusion criteria for this study were established as follows: nurses who provided direct care to premature infants, nurses who were willing to participate in the study, nurses who were available at the time of the study, and nurses with at least one year of experience.

### Sample size

To calculate the sample size for a cross-sectional study that examines the competence of neonatal nurses’ actual practices related to tactile-kinaesthetic stimulation to enhance the oral feeding of preterm infants, we used the following formula: This formula is commonly referred to as the “sample size formula for a single proportion” [25].

$${\rm{n = [z^{\wedge}2*}}\,{\rm{p(1 - p)]/d^{\wedge}2}}$$

where.

n = required sample size.

Z = the standard normal deviation, which is 1.96 for a 95% confidence level.

p = the proportion of neonatal nurses with competent actual practices, which was 0.70 (70%) according to a previous study [26].

d = the desired margin of error, which is 0.05 (5%) for a 95% confidence level.

n = [1.96^2 * 0.70 (1-0.70)]/0.05^2.

n = [3.8416 * 0.21]/0.0025.

*n* = .8067/0.0025.

*n* = 322.68.

**Fig. 1:**
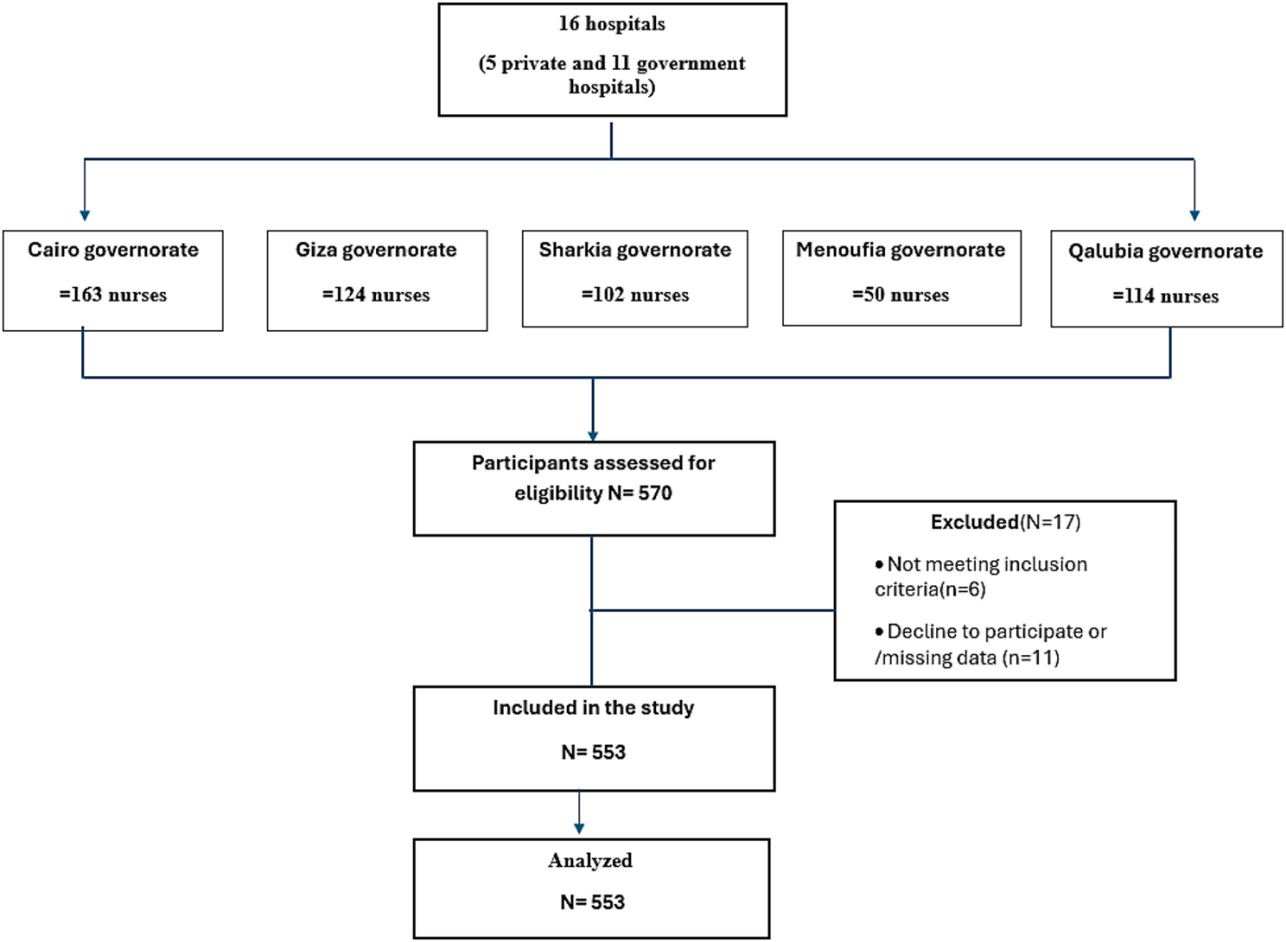
shows the flowdiagram of the included partcipants

Rounding up to the nearest whole number, the required sample size is 323 neonatal nurses. Considering the anticipated attrition of 32 nurses, the final minimum sample size should be 355 neonatal nurses. However, we decided to increase the sample size to 553 to increase the statistical power of the study, enhancing the ability to detect significant effects, as shown in the flow diagram of the included participants (Fig. 1).

### Setting

The study was conducted as a multicenter study in 16 public and private hospitals in five governorates in Egypt from November 2023 to March 2024.

**Fig. 2:**
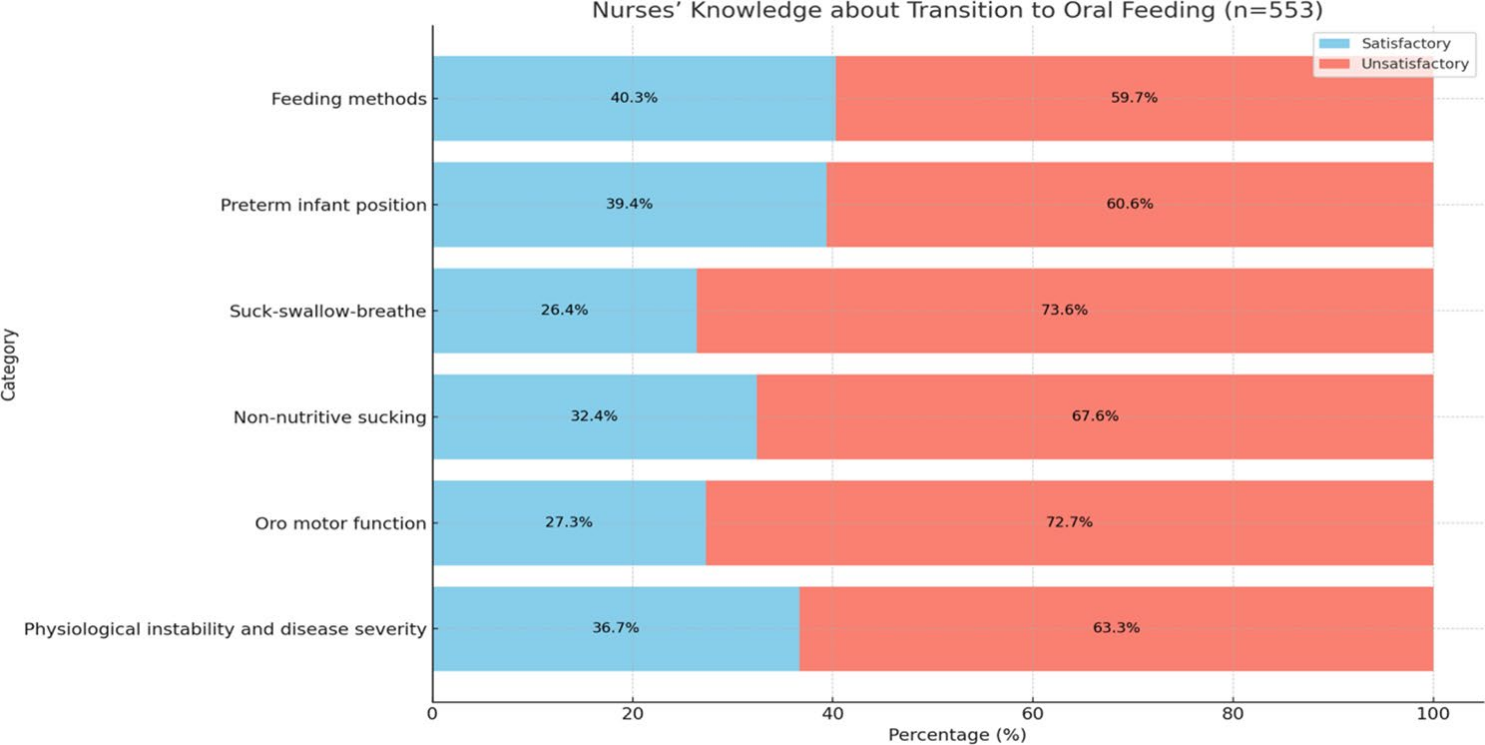
Nurses’ knowledge about transition to Oral Feeding (*n* = 553)

We studied 553 nurses at 5 sites distributed among 16 hospitals, 5 private hospitals and 11 government hospitals. These hospitals were distributed across five governorates as follows: Qalyubia (114 nurses from three hospitals), Sharkia (102 nurses from three hospitals), Menoufia (50 nurses from two hospitals), Cairo (163 nurses from five hospitals), and Giza (124 nurses from three hospitals). All these hospitals have busy neonatal units with approximately 70–90 admissions per month. (Fig. 2).

We selected these hospitals by convenience (including both private and government hospitals), which covered a large number of neonatal nurses working in the NICU. These hospitals, on average, provide care for a large number of premature babies and have a NICU unit with at least 25 incubators.

Selecting hospitals using a random technique involves several steps to ensure that the selection is unbiased and representative. Compile a comprehensive list of all hospitals eligible for selection as mentioned preiously, then Assign a unique number to each hospital on the list. Use a random number generator to select numbers corresponding to hospitals on the list through using online tool that generate random numbers within a specified range. Ensure the number of randomly generated numbers matches the desired sample size of hospitals. Match the randomly generated numbers to the hospitals’ unique identifiers.

### Data collection procedures and study instrument

A self-administered questionnaire was prepared and developed by the researcher in the Arabic language after reviewing recent and relevant literature [14, 21, 27]. Additionally, an English language version has been uploaded as a supplementary file. It consists of the following parts:

### Part I: sociodemographic characteristics

The sociodemographic characteristics of the study participants included age, sex, marital status, education level, qualifications, working experience in NICUs, and previous attendance of any training courses about the feeding transition of premature infants.

### Part II: nurses’ knowledge assessment

This part was concerned with assessing nurses’ knowledge of the transition to oral feeding in preterm infants at NICUs after comprehensively reviewing the related literature [14, 21, 27].

A total of 30 closed-ended (true and false) questions were included. The NRS-2002 was subdivided into five domains: physiological instability and disease severity, oro-motor function, nonnutritive sucking, suck-swallow-breathe, preterm infant position, and feeding pattern and methods. The neonatal nurses answered each item by selecting “true” or “false”.

### Scoring system

The answers were calculated as follows:

The correct answer was given a grade of 1, and the incorrect answer was given a grade of zero. The total grade was 30. The total patient knowledge score was categorized as follows: Patients with a knowledge level greater than or equal to 80% (≥ 24 grade) were considered to indicate a satisfactory level of knowledge. A score less than 80% (0–23 grade) was considered an unsatisfactory level of knowledge [28].

### Tool (II): an observational checklist of the nurse’s practice

This part concerned the assessment of the actual nursing practice regarding the transition to oral feeding in preterm infants at NICUs. It was developed by the researchers after comprehensively reviewing the related literature [19, 24] and consisted of 12 observational checklists related to the transition to the oral feeding process. The neonatal nurses answered each item by selecting “Done correctly”, “Done incorrectly”, or not.

### Scoring system

The answers were calculated as follows:

The items observed to be done correctly were scored “2”, while those done incorrectly were scored “1”, and those not done were scored “0”. The total score on the nurses’ practice checklist (24) was categorized as follows: a score less than 80% was considered unsatisfactory, and a score equal to or more than 80% was considered satisfactory [28].

### Tool (III): nurses’ attitude assessment

The researcher designed the assessment tool based on related literature [14, 21, 27] to evaluate nurses’ attitudes regarding the transition to oral feeding in NICUs. The survey included 12 items, and participants responded on a 3-point Likert scale. The scoring system was classified as follows: “agree” was given a score of 2, “sometimes” was given a score of 1, and “disagree” was given a score of 0. A score of 80% or more reflects a positive attitude, while a score less than 80% reflects a negative attitude [29].

### Procedure

After securing official permission from the designated hospitals and the neonatal intensive care unit (NICU), the fieldwork was conducted over three months, from November 2023 to March 31, 2024. The researchers first introduced themselves to the nurses, explaining the study’s purpose, objectives, and content to gain their cooperation. The authors assured the nurses that their responses would remain anonymous and that the data would be used exclusively for scientific research, maintaining strict confidentiality.

A pilot study involving 55 nurses (10% of the total sample size) was performed to test the clarity, applicability, relevance, and feasibility of the tools and to determine the time needed for data collection. After analyzing the pilot study’s results, the necessary modifications were made. Finally, the nurses involved in the pilot study were subsequently excluded from the study sample.

The data were collected twice a week on Saturdays and Tuesdays during both morning and afternoon shifts at the specified settings until the required sample size was achieved. Each participant underwent a 20-minute individual interview using the nurses’ knowledge assessment and Nurses’ Attitude Assessment tool. Additionally, nurses were observed by the researchers and assessed while performing nursing care for neonates throughout their shifts using observational checklists, which the researchers completed.

### Validity and reliability

The researcher ensured that the questionnaire content and the alignment of the questions addressed the aims, objectives, and research questions of this study. The questionnaire was guided by a comprehensive literature review and assessed for content validity by a panel of five experts who evaluated its representativeness for the target construct.

The reliability of the tool was measured through Cronbach’s alpha test, which yielded the following results: knowledge (α = 0.833), practice (α = 0.891), and attitude (α = 0.870).

### Statistical analysis

The data were analyzed using SPSS version 22.0. The general characteristics were described using descriptive statistics. Spearman’s rank correlation [30] *was used to measure* the strength and direction of *associations* between knowledge, *attitudes*, and practices. Binary logistic regression analysis identified several significant predictors of the dependent variable “Total Practice” [30]. A binary logistic regression model was used to examine the relationship between ‘Total Practice’ and a set of independent variables. The model includes the following independent variables: age, years of experience, sex, education level, marital status, training courses, nurse-to-neonate ratio, employment status, perception of workload, total knowledge, and total attitude. The ‘Total Practice’ variable is the dependent variable and was initially coded as satisfactory or unsatisfactory; this variable was recoded as binary values (1 and 0) for logistic regression analysis. The receiver operating characteristic (ROC) curve generated for the binary logistic regression model demonstrated the model’s ability to distinguish between the two classes (“satisfactory” vs. “unsatisfactory” total practice). The area under the curve ranged from 0.5 (random model) to 1 (perfect model) [30]. Higher AUC values indicate better model performance. *P* < .05 indicated statistical significance.

## Results

Table 1 presents the characteristics of the 553 nurses who participated in the study. The majority of the nurses (39.6%) were aged between 30 and 40 years, with a mean age of 33.67 years (SD = 7.3). In terms of experience, 36.2% of the nurses had between 7 and 13 years of experience, with a mean of 10.37 (SD = 5.5) years. Regarding sex, 55.7% of the participants were female, and 44.3% were male. The educational level of the nurses varied, with 44.8% having a technical health institution degree, 29.3% holding a Bachelor of Nursing degree, and 25.9% having a diploma. Most of the nurses (61.7%) were married, while 38.3% were unmarried. Slightly more than half of the nurses (56.1%) reported having attended training courses about feeding transition, while 43.9% had not. The nurse-to-neonate ratio varied, with 42% of nurses caring for two neonates, 39.2% caring for three neonates, and 18.8% caring for one neonate. Additionally, 65.6% of the nurses were employed full-time, and 34.4% were employed part-time. Finally, 69.6% of the nurses perceived their workload as overloaded, while 30.4% considered their workload to be balanced.

**Table 1:** Characteritsics of studied nurses (*n* = 553)

	*n*	%
Age: 20 -<30 30 - <40 40–50 Mean (SD). 33.67 (7.3)	196 219 138	35.4 39.6 25
Years of experience: 1 - <7 7 - <13 13–20 Mean (SD). 10.37 (5.5)	174 179 200	31.5 32.4 36.2
Gender: Male Female	245 308	44.3 55.7
Education level: Diploma Technical health inistitute Bachelor of nursing	143 248 162	25.9 44.8 29.3
Marital status: Married Unmarried	341 212	61.7 38.3
Training courses about feeding transition: Yes No	243 310	56.1 43.9
Nurse to neonate ratio: 1:1 1:2 1:3 Mean (SD). 2.2 (0.73)	104 232 217	18.8 42 39.2
Employment status: Fulltime Parttime	363 190	65.6 34.4
Perception of workload: Overload Balanced	385 168	69.6 30.4

Figure 2 presents the nurses’ knowledge about various aspects related to the transition to oral feeding in preterm infants. The results indicate that a significant proportion of nurses had unsatisfactory knowledge in several areas. Specifically, 72.7% of the nurses had unsatisfactory knowledge about oro-motor function, 73.6% lacked adequate knowledge about the suck-and-swallow pattern, and 67.6% had unsatisfactory knowledge about nonnutritive sucking. Additionally, 63.3% of the nurses demonstrated unsatisfactory knowledge regarding physiological instability and disease severity, while 60.6% had unsatisfactory knowledge about the appropriate position for preterm infants during feeding. However, 59.7% of the nurses provided satisfactory knowledge about the feeding methods. These findings suggest that a substantial number of nurses in this study had knowledge gaps in various aspects related to the transition to oral feeding in preterm infants, which could impact their ability to provide optimal care and support during this critical phase.

Table 2 presents the overall knowledge, practices, and attitudes of the 553 nurses regarding the transition to oral feeding in preterm infants. The results showed that 64.6% of the nurses had unsatisfactory total knowledge, while only 35.4% demonstrated satisfactory knowledge. In terms of practice, 58.6% of the nurses reported unsatisfactory practices, and 41.4% had satisfactory practices related to the transition to oral feeding. Regarding attitudes, 55% of the nurses exhibited a positive attitude, while 45% had a negative attitude toward this aspect of care. These findings suggest that a significant proportion of nurses in the study had knowledge deficits and suboptimal practices concerning the transition to oral feeding in preterm infants. However, it is encouraging to note that more than half of the nurses had a positive attitude, which could facilitate the implementation of targeted educational interventions and support measures to improve their knowledge and practices in this crucial area of neonatal care.

**Table 2:** Correlation between nurses’ knowledge, practice, and attitude (*n* = 553)

	*n*	%	Knolwedge	Practice	Attitude
Total knowledge: Satisfactory Unsatisfactory	196 357	35.4 64.6		r.260 p.000	r.215 p.000
Total practice: Satisfactory Unsatisfactory	229 324	41.4 58.6	r.260 p.000		r.156 p.000
Total attitude: Positive Negative	304 239	55 45	r.215 p.000	r.156 p.000	

Additionally, correlations between the total knowledge, total practice, and total attitude scores of the nurses regarding the transition to oral feeding in preterm infants were calculated. The results indicate a statistically significant positive correlation between total knowledge and total practice (*r* = .260, *p* < .001), suggesting that nurses with higher knowledge levels tended to practice better related to this aspect of care. Similarly, a significant positive correlation was observed between total knowledge and total attitude (*r* = .215, *p* < .001), indicating that nurses with greater knowledge were more likely to have a positive attitude toward the transition to oral feeding. Furthermore, a significant positive correlation was found between total practice and total attitude (*r* = .156, *p* < .001), suggesting that nurses with more satisfactory practices tended to exhibit a more positive attitude.

Based on the binary logistic regression analysis (Table 3), several coefficients were found to be statistically significant, indicating that they have a meaningful impact on the likelihood of achieving a “satisfactory” total practice rating. Males are significantly more likely to achieve a “satisfactory” rating than females are, as indicated by a positive coefficient (0.449) with a p value of 0.027. Individuals with a diploma or education from a health institution are less likely to have a “satisfactory” rating than are those with a bachelor’s degree, with negative coefficients of -1.127 and − 0.764 and p values of 0.00007 and 0.0015, respectively. Unmarried individuals are also less likely to achieve a “satisfactory” rating, as shown by a coefficient of -0.679 and a p value of 0.0025. A nurse-to-neonate ratio of three or two significantly decreased the likelihood of a “satisfactory” rating compared to a ratio of one, with coefficients of -0.857 and − 0.876 and p values of 0.0028 and 0.0017, respectively. Part-time employment significantly increases the likelihood of achieving a “satisfactory” rating, with a coefficient of 0.932 and a p value of 0.000019. Finally, individuals with unsatisfactory knowledge are less likely to achieve a “satisfactory” rating, as indicated by a coefficient of -0.929 and a p value of 0.000013, while a positive attitude significantly increases the likelihood of achieving a “satisfactory” rating, with a coefficient of 0.529 and a p value of 0.0102. These significant coefficients highlight the factors that most strongly influence the outcome, and by understanding these relationships, actionable steps can be taken to improve the likelihood of achieving a “satisfactory” total practice rating.

**Table 3:** Binary logistic regression analysis for nurses’ practice level

Variable	Coefficient	Std. Error	z-value	*p*-value	[0.025, 0.975]
const	0.9861	0.712	1.384	0.166	[-0.410, 2.382]
Age	-0.0089	0.022	-0.403	0.687	[-0.052, 0.034]
Years of Experience	0.0258	0.029	0.905	0.365	[-0.030, 0.082]
Gender_Male	0.4486	0.203	2.215	0.027	[0.052, 0.846]
Education Level_Diploma	-1.1265	0.283	-3.976	0.000	[-1.682, -0.571]
Education Level_Health Institute	-0.7637	0.241	-3.172	0.002	[-1.236, -0.292]
Marital Status_Unmarried	-0.6787	0.224	-3.024	0.002	[-1.119, -0.239]
Training Courses_Yes	0.3958	0.209	1.895	0.058	[-0.014, 0.805]
Nurse-to-Neonate Ratio_Three	-0.8569	0.286	-2.994	0.003	[-1.418, -0.296]
Nurse-to-Neonate Ratio_Two	-0.8757	0.279	-3.136	0.002	[-1.423, -0.328]
Employment Status_Part	0.9318	0.218	4.272	0.000	[0.504, 1.359]
Perception of Workload_Overload	-0.2535	0.222	-1.142	0.254	[-0.689, 0.182]
Total Knowledge_UnSatisfactory	-0.9291	0.213	-4.364	0.000	[-1.346, -0.512]
Total Attitude_Positive	0.5290	0.206	2.568	0.010	[0.125, 0.933]

**Fig. 3:**
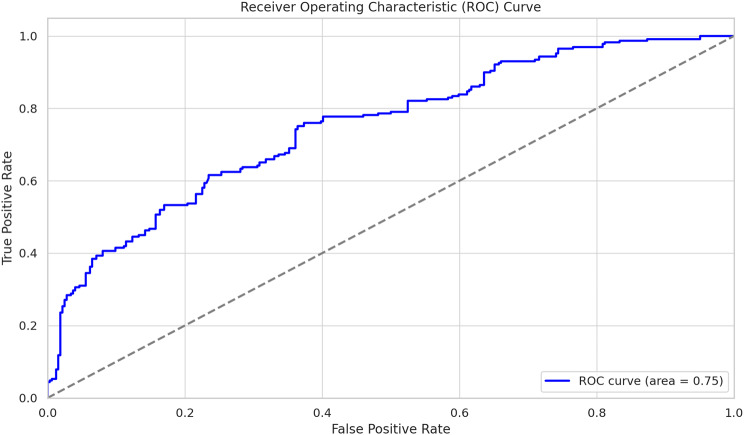
Receiver operating characteristics (ROC) Curve

The ROC curve generated from the model had an AUC value of approximately 0.76, suggesting that the model has good performance in distinguishing between the two classes. An AUC of approximately 0.76 indicates that the model has good discriminatory ability, making it a reliable tool for predicting “Total Practice” as either “satisfactory” or “unsatisfactory”, Fig. 3.

## Discussion

The findings from this cross-sectional study underscore significant gaps in the knowledge, practice, and attitudes of nurses working in the NICU regarding the transition to oral feeding among preterm infants. The current results indicate that a substantial proportion of nurses lack adequate knowledge in several critical areas related to this transition. Specifically, the majority of nurses demonstrated unsatisfactory knowledge of oro-motor function, the suck-swallow-breathe pattern, and nonnutritive sucking. Additionally, a significant percentage of nurses exhibited insufficient understanding of physiological instability and disease severity. However, 59.7% of the nurses displayed satisfactory knowledge regarding feeding methods.

These knowledge deficits are concerning because they could hinder neonatal nurses’ ability to provide optimal care and support during the critical-to-oral-feeding transition among preterm infants. Effective management of this transition requires comprehensive understanding and skills in these key areas to guarantee that preterm infants receive optimal care and achieve effective attainment regarding oral feeding.

These study findings are consistent with previous research by Aykanat and Gözen [14], who reported in their descriptive, cross-sectional study that the correct responses were particularly low for items associated with cue-based feeding, intervention to promote oral-motor development, nonnutritive sucking, and infant positioning during oral feeding. These findings align with our findings that a majority of nurses lack sufficient knowledge in these crucial areas. This similarity is likely due to the study being conducted under similar conditions. Also, supported by Buloze [31] conducted a study on nurses with similar characteristics and reported that most nurses had acceptable knowledge related to nonnutritive sucking and feeding methods, supporting our findings. Furthermore, Girgin et al. reported that a majority of neonatal nurses lacked knowledge related to cue-based feeding interventions, oral motor stimulation, and positioning techniques [24]. These results may be due to the fact that most nurses did not attend training courses previously about feeding transition, similar to our study. In a qualitative study by Brantes and Curado [32], neonatal nurses recognized the importance of continuing education and training in assessing oral feeding skills.

Throughout our study, we evaluated nurses’ knowledge, practices, and attitudes regarding oral feeding transition. Approximately two-thirds of the nurses had unsatisfactory total knowledge, highlighting the need for educational interventions. The practices of these nurses also mirrored this trend, with 58.6% of the nurses reporting unsatisfactory practices. This indicates a clear gap in the application of knowledge to practice, which is crucial for effective patient care. Despite these challenges, it is encouraging to note that more than half of the nurses exhibited a positive attitude concerning the transition to oral feeding.

The majority of nurses’ positive attitudes are promising indicators. An individual’s attitude can have a significant impact on his or her willingness to engage in educational opportunities and improve practices. This positive disposition could be leveraged to implement targeted educational programs designed to address knowledge and practice gaps. Such interventions could foster an environment of continuous learning and improvement, ultimately enhancing the quality of care provided to preterm infants [33].

These results are supported by a quasi-experimental single-group study by Naz et al. [12], which studied 36 nurses. The majority of them had a diploma in nursing, 4–10 years of experience, and belonged to the 31–45 years age category, which aligns with our nurses’ age and experience. The study showed that the majority of the nurses practiced incompetent practices prior to training.

Similarly, Çelen et al. [19] reported that most nurses had poor practices in supporting oral feeding in preterm infants before any intervention. These results are supported by the finding that more than half of the nurses did not receive education on preterm infant feeding, similar to our results. Also, Buloze [31] conducted a study on nurses with similar characteristics and stated that nurses working in tertiary referral hospitals have shown an acceptable level of knowledge and a positive attitude related to the feeding transition of infants with low birth weight. Another qualitative study conducted by Astuti et al. [34] concluded that neonatal nurses play a pivotal role in enhancing OFP among premature infants. A descriptive study in Egypt highlighted that nurses’ knowledge of trophic feeding practices is essential for improving preterm infant outcomes [35]. In addition, nurses must be skilled in developmental stimulation to enhance oral feeding readiness in preterm infants [36].

Furthermore, the current results highlight the interconnected nature of knowledge, practices, and attitudes among nurses, as evidenced by the correlations between the studied variables. The significant positive correlations between total knowledge and total practice (*r* = .260, *p* < .001), total knowledge and total attitude (*r* = .215, *p* < .001), and total practice and total attitude (*r* = .156, *p* < .001) emphasize the importance of a holistic approach to education and training. Enhancing knowledge is likely to lead to improved practices and more positive attitudes, creating a virtuous cycle that benefits both nurses and their patients.

These findings suggest that improving knowledge through education could directly enhance clinical practices and highlight the importance of knowledge in shaping attitudes, which in turn can influence practices. Additionally, improving practices can reinforce positive attitudes, creating a supportive environment for ongoing learning and improvement [37]. Similarly, Girgin et al. [22] reported that providing training to nurses on the transition to oral feeding in infants increased knowledge level. Nursing intervention support is needed in caring for infants with delayed oral feeding skills through teamwork among nurses, families and parents [34]. Moreover, before conducting an education program, Beissel et al. [38] indicated that nurses’ performance related to oral feeding was poor. Additionally, Salah and Hassan [39] showed a positive correlation between nurses’ knowledge, practices and attitudes, reinforcing the interconnectedness of these elements in clinical settings. This similarity is likely due to the study being conducted in the same geographic area and involving nurses with similar characteristics.

The results of the binary regression analysis provide compelling evidence that several key factors significantly influence the practice levels of neonatal nurses regarding the transition to oral feeding in preterm infants. The model demonstrated a good fit for the data, reinforcing the validity of the findings (it has an AUC value of approximately 0.76, suggesting that it has a good level of performance). Males are significantly more likely to achieve a “satisfactory” rating than females are, suggesting a sex disparity in practice quality. Additionally, we highlight the importance of higher education in achieving better practice outcomes. A nurse-to-neonate ratio of three or two significantly decreases the likelihood of a “satisfactory” rating compared to a ratio of one, underscoring the importance of adequate staffing levels for better practice outcomes. Part-time employment significantly increases the likelihood of achieving a “satisfactory” rating, suggesting potential differences in practice quality between full-time and part-time practitioners. Nurses with unsatisfactory knowledge are less likely to achieve a “satisfactory” rating, while a positive attitude significantly increases the likelihood of achieving satisfactory knowledge and attitude in practice quality.

These findings are consistent with previous studies. Salah and Hassan [39] noted that a positive attitude significantly improves practice levels. Abo El Magd Fathi et al. [26] who conduct study in Egypt as our study, emphasized that higher education levels and increased nursing experience enhance practice levels related to sensorimotor stimulation to improve oral feeding in preterm infants. Similarly, Naz et al. [12] reported that attending training courses boosts nurses’ skills. Girgin and Gözen [14] highlighted that a heavy workload negatively impacts nurses’ skills, underscoring the importance of a supportive working environment. The success of achieving oral feeding readiness is influenced by the therapeutic care environment and caregiver competence [40]. Additionally, Li et al. [41] reported that caregivers’ knowledge improves their oral feeding skills for preterm infants with dysphagia. Nurses are vital for effectively improving preterm oral feeding readiness, as they provide efficient nursing care to improve the oral feeding transition power of infants [38].

## Conclusion

This study highlights significant gaps in the knowledge, practice, and attitudes of neonatal nurses regarding the transition to oral feeding in preterm infants. The findings revealed that a substantial proportion of nurses lacked adequate knowledge of critical areas, such as oro-motor function, the suck-swallow-breathe pattern, and nonnutritive sucking. These knowledge deficits potentially hinder the ability of nurses to provide optimal care during this crucial transition. However, the generally positive attitude among nurses suggests a strong foundation for implementing targeted educational interventions to enhance their knowledge and practices, ultimately improving care for preterm infants.

Addressing these gaps through comprehensive educational programs and ongoing support will be essential for ensuring high-quality care for preterm infants during this vital phase of their development.

### Strengths of the study

The study covers multiple hospitals across various regions, providing a broad perspective on the performance of neonatal nurses. The use of self-administered questionnaires and observational checklists ensures a thorough assessment of nurses’ knowledge, practices, and attitudes that improve data collection. This study employs robust statistical methods, including binary logistic regression, to identify significant predictors of satisfactory practices, ensuring the reliability of the findings.

### Limitations of the study

The cross-sectional design limits the ability to establish causality between identified gaps in knowledge and observed practices. Self-Selection Bias: nurses who chose to participate in the study might have a greater interest or proficiency in the subject, leading to results that are not fully representative of the broader nurse population.

### Implications for practice and education

Develop policies that mandate regular training and assessments to ensure that neonatal nurses remain up-to-date with best practices in oral feeding transition. A supportive work environment with manageable workloads and adequate nurse-to-patient ratios should be ensured. Ongoing professional development for neonatal nurses should be encouraged through workshops, seminars, and online courses.

## Electronic supplementary material

Below is the link to the electronic supplementary material.

Supplementary Material 1

## Data Availability

Data is provided within the manuscript or supplementary information files.

## Abbreviations

WHO: World Health Organization
NICU: Neonatal Intensive Care Unit
GA: Gestational Age
