# Supplementary material for: Assessing neonatal nurses: transitioning preterm infants to oral feeding - a multicenter cross-sectional study
Source: BMC Nurs. 2025 Jan 9;24:32. doi: 10.1186/s12912-024-02647-9 (PMC11715433; doi:10.1186/s12912-024-02647-9)
Supplement: Supplementary file 1 — Supplementary Material 1 [file 12912_2024_2647_MOESM1_ESM.docx]

**Appendix I**

1. **Socio-demographic data:**

**1- Age** ( )

**2-Gender** ( )

**3-Educational Background/ *Academic Qualifications***

1. Nursing Diploma
2. Technician Institute of Nursing
3. Bachelor's degree BSN
4. Master MSN
5. Doctorate

**4-** **Employment status**

1. Fulltime
2. Part-time

**5**- **Marital Status**

1. Married 🞎
2. Separated 🞎
3. Divorced 🞎
4. Widowed 🞎

**6- Specialized training course attendance:**

(1) Yes

(2) No

**9- Year of experience:……….**

**10-Perception of workload:**

1. Balanced workload
2. Overload

**11-Nurse's/ neonate Ratio:**

1. 1: 1
2. 1: 2
3. 1: 3

**Appendix II**

**Nurses’ knowledge, Practice and Attitude**

- **Nurses’ knowledge**

| **No** | **Questions** | **True** | **False** |
| --- | --- | --- | --- |
| **physiological instability and disease severity:** | | | |
|  | Physiological stability measures assessment is standard practice before oral feeding in preterm infants. |  |  |
|  | Monitoring of physiological measures for each participant was not altered from physician-prescribed care. |  |  |
|  | Neonatal nurses are familiar with the signs and symptoms of physiological instability in preterm infants. |  |  |
|  | The transition to oral feeding and oral feeding readiness of the preterm infant is significantly influenced by the severity of the disease. |  |  |
|  | Oral feeding readiness of the preterm infant is based on neurologic maturation. |  |  |
| **Oro motor function:** | | | |
|  | Coordination between the autonomic, motor, and self-regulation systems of the preterm infant develops with sufficient neurological maturity |  |  |
|  | As the postmenstrual age of a preterm infant increases, neurological maturity decreases. |  |  |
|  | Oral–motor interventions consist of 10 min of only lip and gum stimulation. |  |  |
|  | Oral–motor interventions should be implemented when the preterm infant has transitioned to full oral feeding. |  |  |
|  | Prolonged gavage feeding delays the development of oral–motor skills. |  |  |
|  | Prolonged tube feeding slow the oral-motor function progression. |  |  |
| **Non-nutritive sucking:** | | | |
|  | In non-nutritive sucking, sucking durations are long and rhythmic. |  |  |
|  | Non-nutritive sucking has no effect on reducing the duration of gavage feeding. |  |  |
|  | Non-nutritive sucking is only done with a pacifier. |  |  |
|  | Non-nutritive sucking is done with the mother's breast, preferably after pumping. |  |  |
|  | Non-nutritive sucking has a serious effect on oxygen saturation levels after using a pacifier. |  |  |
|  | Non-nutritive sucking has no impact on the heart rate after using a pacifier. |  |  |
|  | The stability of physiologic functions and rhythmic non-nutritive sucking are prerequisites for beginning the transition to oral feeding. |  |  |
|  | Hand or fist sucking is one of the infant’s signs of hunger. |  |  |
| **suck-swallow-breathe:** | | | |
|  | In a coordinated feed, there is a 1:1:1 suck-swallow-breathe ratio |  |  |
|  | Immature suck-swallow-breathe coordination emerges at 32 weeks of gestation. |  |  |
|  | Hand or fist sucking is one of the cues that indicate a preterm infant is ready for oral feeding. |  |  |
| **Preterm infant position:** | | | |
|  | The recommended position for bottle feeding a preterm infant is the semi-elevated supine position, in which the nurse holds the baby in her lap with the head and neck elevated to 45–60 degrees. |  |  |
|  | There is no difference between using the semi-elevated supine or semi-elevated side-lying positions while feeding in terms of preterm infant's vital signs. |  |  |
|  | Due to the effect of hydrostatic pressure in the semi-elevated supine position, a large volume of milk from the bottle increases the infant's wakefulness and shortens feed duration. |  |  |
|  | Feeding the preterm infant in a semi-elevated side-lying position raises the likelihood of aspiration. |  |  |
|  | It is recommended for all preterm newborns to be fed in a semi-elevated side-lying position, irrespective of their condition. |  |  |
|  | Feeding in a semi-elevated side-lying posture is not recommended for premature infants with gastroesophageal reflux. |  |  |
| **feeding methods:** | | | |
|  | During the process of transitioning to oral feeding, the greater number of bottle feeds shortens the time required for the transition to full oral feeding. |  |  |
|  | After a feed, the remaining breast milk in the bottle can be put in the refrigerator and reused within 3 days. |  |  |
|  | In bottle-fed infants, twisting or moving the nipple back and forth in the mouth during feeds stimulates feeding and increases feeding success. |  |  |
|  | Cup feeding absolutely increases the frequency of breastfeeding after discharge. |  |  |
|  | Feeding with a cup/bottle/spoon/syringe is an alternative feeding method used to deliver expressed breast milk when the mother is not able to come to the neonatal intensive care unit. |  |  |

- **Nurses ‘practice:**

| **No** | **Questions** | **Done correctly** | **Done incorrectly** | **Not done** |
| --- | --- | --- | --- | --- |
|  | Measure the physiological stability parameters before, during and after the feeding:   - Heart rate - Respiratory rate - Oxygen level - Prevent aspiration. - Color change |  |  |  |
|  | Early introduction of oral stimulation:   - Checking Body in a Flexed Position - Checking Awake State - Checking Energy for Feed - Checking Behavioral Stress Cues during Feeding |  |  |  |
|  | Facilitate Non-nutritive Suck:   - breast - pacifier - Fingers |  |  |  |
|  | Sensorimotor interventions:   - Preparations before initiation of sensorimotor stimulation to preterm infants - Oral stimulation (perioral and intraoral stimulation) combined with non-nutritive sucking. - Tactile kinaesthetic stimulation |  |  |  |
|  | Assess the behavioral cues of the preterm infant:   - Readiness cues - Stress cues |  |  |  |
|  | Maintain the appropriate preterm infant positioning:   - Semi-elevated side-lying position in lap - Semi-elevated side-lying position in incubator - Semi-elevated supine position in incubator |  |  |  |
|  | Monitor coordinated suck-swallow-breathe during feeding:   - Maintains a rhythmic feeding pattern. - Ratio of suck, swallow, and breathing = 1:1:1 - Duration and pattern of the sucking cycle - Lip closure reaction when nipple or bottle teat enters the mouth |  |  |  |
|  | Monitor the Nipple used for bottle feeding:   - Standard Flow - Slow Flow (green nipple or yellow nipple) - Moderate-flow nipple - Fast-flow nipple |  |  |  |
|  | Observe the behavioral state of the preterm infant:   - **Sleep:** eyes closed, regular or irregular respirations. - **Drowsy:** eyes opening and closing, slight movement of face or limbs. - **Awake:** eyes open, no movement to full body movement. - **Fussy/crying**: eyes open or closed, audible whimper to cry, - **Color change.** - **Rooting** - **Lips pursed.** - **Hands-to-mouth mouthing** - **Smacking Lips** |  |  |  |
|  | Provide a calm, quiet area, dim lighting, no distractions, and a restful environment between feedings. |  |  |  |
|  | Monitor infant body weight before and after feeding. |  |  |  |
|  | Preparation and steps of milk storage:   - Refrigerator - Room temperature - Valid duration |  |  |  |
|  | assist the parents in interacting with their infant and understanding the behaviors. |  |  |  |
|  | Confirm parental caregiver ability to support satisfactory oral feeding prior to hospital discharge |  |  |  |

- **Nurses Attitude:**

| **No** | **Questions** | **Agree** | **Disagree** |
| --- | --- | --- | --- |
|  | Recognizing the importance of early feeding skills assessment to support preterm infants during their transition to oral feeding and Identifying problem areas is based on the nurse's responsibility. | **✓** |  |
|  | During infant feeding, actively managing sources of light and sound and eliminating unnecessary distractions can foster a calm and soothing environment conducive to nurturing feeding experiences. | **✓** |  |
|  | transition to direct breast-feeding during his/her stay in the NICU has a higher chance of continued breastfeeding at 6 months of age | **✓** |  |
|  | cue-based feeding is more beneficial and plays a major role in feeding readiness in preterm infants. | **✓** |  |
|  | non-nutritive sucking plays an essential role in improving the maternal bonding | **✓** |  |
|  | Infant formula is an ideal option in transition to oral feeding for premature infants in the NICU. |  | **✓** |
|  | Overcoming challenges to breastfeeding promotion in the NICU is very important to facilitate optimal infant nutrition and improve long-term health outcomes for preterm infants. | **✓** |  |
|  | Understanding that swallowing and breathing coordination stops to mature after 6 months of birth. |  | **✓** |
|  | introduction of nonnutritive sucking should be held until the preterm infant reaches a certain gestational age |  | **✓** |
|  | assist the parents in interacting with their infant and understanding the behaviors increase nurse’s workload |  |  |
|  | breastfeeding takes priority over bottle feeding when a mother wishes to breastfeed exclusively. | **✓** |  |
|  | Encouraging the parents to learn about infant feeding cues, such as the rooting reflex and sucking on their fingers, is not mandatory. |  | **✓** |
